# Ocular myasthenia gravis saccades as a measure of extraocular muscle function

**DOI:** 10.3389/fopht.2022.938088

**Published:** 2022-10-12

**Authors:** Sui H. Wong, Matthew James Bancroft, Vijay K. Tailor, Mohamed Abbas, Akila Sekar, Claire Noble, Maria Theodorou, Diego Kaski

**Affiliations:** ^1^ Moorfields Eye Hospital, London, United Kingdom; ^2^ Department of Clinical and Movement Neurosciences, University College London, London, United Kingdom; ^3^ Department of Experimental Psychology, University College London, London, United Kingdom

**Keywords:** ocular myasthenia gravis, extraocular muscle, myasthenia gravis, saccades, video-oculography

## Abstract

**Background:**

It is important to understand the pathophysiology of ocular myasthenia gravis (OMG) to improve treatment.

**Aim:**

To use modern video-oculography to characterise saccadic eye movements in patients with OMG, including anti-AChR, anti-MuSK, anti-LRP4, and seronegative OMG.

**Methods:**

In total, 21 patients with OMG and five age-matched healthy control subjects underwent video-oculography. Participants performed a sequence of horizontal saccades (3 minutes each) at ±5°, ± 10°, and ±20°, followed by 3 minutes of saccades directed at randomly presented targets at ±5°, ± 10°, and ±15°. We recorded the direction, amplitude, duration, peak, and average velocity of each saccade for each task for each participant.

**Results:**

Saccadic amplitude, duration, and average velocity were all lower in OMG patients than in control subjects (*p* < 0.021). Saccadic amplitude and velocity decreased over time, but this decrease was similar in OMG patients and control subjects. Fixation drift and ocular disparity tended to be greater in OMG patients than in control subjects. Saccadic intrusions occurred more frequently in OMG patients than in control subjects (*p* < 0.001). No significant effects of time or group by time on fixation drift or ocular disparity were found.

**Discussion:**

Saccadic velocities in OMG patients differed from those in normal control subjects, which suggests that OMG affects fast-twitch fibres, although fast-twitch fibres were still able to generate “twitch” or “quiver” movements in the presence of even severe ophthalmoplegia. Slow-twitch muscle fibres involved in gaze holding were also affected, accounting for increased fixation drift following saccades. Our objective finding of increased fixation drift and a larger number of saccadic intrusions mirror our anecdotal experience of patients with OMG who report significant diplopia despite minimal ophthalmoplegia on examination. Such microsaccades may be a surrogate for compensation of a gaze-holding deficit in MG.

## Introduction

Myasthenia gravis (MG) is an autoimmune disorder of the neuromuscular junction that presents initially with ocular symptoms in 80%–85% of patients (1, 2). Up to 40%–70% of these patients may have ocular symptoms only (1, 3), i.e., ocular myasthenia gravis (OMG). The ocular symptoms of diplopia and ptosis can be disabling and adversely affect a patient’s quality of life (4). Therefore, an understanding of the pathophysiology underlying these ocular symptoms is important for the advancement of treatment and development of patient-centred care in MG.

The hallmarks of MG are the variability of symptoms and signs of neuromuscular weakness. Classical ocular symptoms of MG (5) include the Cogan’s lid twitch and “quiver”, or twitch-like movements in paretic muscles (6). Although “lid twitch” is not specific to MG (7), a prerequisite of Dr. Cogan’s original definition of MG is the presence of ptosis. This suggests a differential impact of MG in the extraocular muscle (EOM) fibres, with relative preservation of fast-twitch muscle fibres.

Previous studies of eye movement in MG (i.e., not limited to OMG) have shed some light on the pathophysiological effects of MG on ocular muscles, e.g., preservation of high-velocity saccades in the presence of severe ophthalmoplegia, indicating selective impairment of tonic fibres (8, 9), and variability of saccades (10, 11). However, there remains much to be elucidated, as EOMs contain six muscle fibre types distributed according to the different layers and are recruited at different phases of eye movements (12, 13).

In addition, mapping eye movement studies with the clinical phenotype may be helpful to further understand the pathophysiology of OMG. The pathogenic antibodies in MG include antibodies against the acetylcholine receptor (anti-AChR), muscle-specific kinase (anti-MuSK), and low-density lipoprotein receptor-related protein 4 (anti-LRP4). However up to 40%–50% of patients with OMG are seronegative, of whom approximately 50% have been found to produce anti-AChR antibodies; more recently, it has been found that a smaller percentage produce anti-MuSK and anti-LRP4 antibodies (14, 15). The clinical observation that OMG patients who produce anti-AChR antibodies have more severe ophthalmoplegia and ptosis than seronegative OMG patients suggests that different types of EOM muscle fibres are affected (unpublished observation, SHW).

This preliminary study aims to characterise saccadic eye movements across all OMG phenotypes, including anti-AChR, anti-MuSK, anti-LRP4, and seronegative OMG, with a focus on fixational eye movement abnormalities.

## Methods

### Participants and apparatus

Twenty-one participants with MG (11 with anti-AChR OMG, 1 with anti-LRP4 OMG, 1 with anti-MuSK OMG, 7 with seronegative OMG, and 1 with anti-AChR secondary generalised MG) were prospectively identified and recruited from the OMG clinic at Moorfields Eye Hospital NHS Foundation Trust. All participants underwent a full clinical examination (orthoptics, neuro-ophthalmology, and general medical assessment) to confirm their diagnosis. Ten control participants were recruited from staff members at Moorfields Eye Hospital NHS Foundation Trust and University College London. After providing informed written consent, participants underwent infrared video-oculography using an EyeLink 1000 eye tracker (SR Research, Ottowa, ON, Canada). We recorded the position of both eyes (where possible) at a sampling rate of 500 Hz while participants performed a range of saccadic tasks. Stimuli were presented on an Eizo Flexscan EV2736W LCD monitor, with 2560 × 1440 pixel resolution, 60 Hz refresh rate, and a physical panel size of 59.7 cm × 33.6 cm. The monitor was calibrated using a Minolta photometer, with luminance linearised by software to give a maximum of 150 cd/m^2^. Participants were positioned with their heads lying on a chin rest, and a forehead bar was applied to minimise head movement. The viewing distance was 75 cm. Stimulus presentation was binocular for all participants and monocular when a large degree of strabismus was present and the eye tracker was unable to record the fellow eye. Participants requiring refractive correction wore their usual spectacles.

### Stimuli and procedures

Before gaze data were recorded, participants performed a binocular five-point calibration, where possible. If this was not possible, monocular calibration of the dominant eye was carried out.

Participants performed the following sequence of saccade tasks, each of which lasted for 3 minutes: horizontal ±5°, vertical ±5°, horizontal ±10°, vertical ±10°, horizontal 20°, vertical 20° (20° steps between ±10° locations), followed by 3 minutes of horizontal saccades with randomly presented targets at ±5°, ± 10°, and ±15°, and 3 minutes of vertical saccades with randomly presented targets at ±5° and ±10°. A red circle subtending approximately 0.5° of visual angle was used as the target stimulus on a mid-grey background. The target was shown for 3 seconds at each location. Participants were allowed to rest for an adequate period between each block of trials (minimum rest time of 60 seconds). All procedures were approved by the Wales REC 6 Research Ethics Committee (IRAS 279233).

### Data analysis

The EyeLink 1000 eye-tracking software parses the gaze data into saccades and fixations using a saccade-picking algorithm. Saccade onset is defined as the point at which velocity exceeds a threshold of 30° per second or the point at which acceleration exceeds a threshold of 8000°/s^2^, and saccade offset is defined as the point at which velocity falls below the threshold. Saccade onset defines fixation offset and vice versa. Data Viewer software (version 4.2.1, SR Research) was used to output the direction, amplitude, duration, peak, and average velocity of each saccade for each task and each participant. Saccades of <2°, saccades that contained blinks, and saccades that had a greater vertical than horizontal amplitude were excluded from the main analysis. In this way, all leftward/rightward saccades of >2° were examined, irrespective of their relationship with the target step.

For each task, saccades were grouped into three time bins, corresponding to the first, second, and third minute of the tasks.

To examine fixation stability, two separate analyses were performed. In the first analysis, monocular data were used, and the horizontal difference in gaze location at the start and end of each fixation of >100 ms in duration was taken as a measure of drift. In the second analysis, binocular data (available from 17/21 patients and all controls) were used, and the difference in average gaze location between the left and the right eyes during all fixations was taken as a measure of ocular disparity. Drift and ocular disparity were reported in degrees of visual angle.

Square wave jerks (SWJs), a type of saccadic intrusion, were defined as saccades of <2° that were followed within 300 ms by another saccade of <2° in the opposite direction (Leigh and Zee, 2006). The number of SWJs across all tasks was counted for each participant.

Data were statistically analysed in SPSS 28.0 (IBM Corp.). Fixation stability and saccadic metrics were analysed by repeated-measures ANOVA, with the between-subject factor group (OMG vs. control) and the within-subject factor of time (first vs. second vs. third minute). Greenhouse–Geisser correction was used when Mauchly’s test of sphericity was significant. The difference between groups (OMG vs. control) in the number of SWJs was analysed by Mann–Whitney *U*-test owing to data deviating from normality. Identical results were obtained when normalising the number of SWJs by the total number of saccades; non-normalised data are reported for ease of interpretation. Statistical significance was set at 0.05 after correction for multiple comparisons using Bonferroni adjustment. Summary statistics are presented as mean ± 1 SE for fixation stability and saccadic metrics, and median ± interquartile range (IQR) is presented for SWJs.

## Results

**Figure 1:**
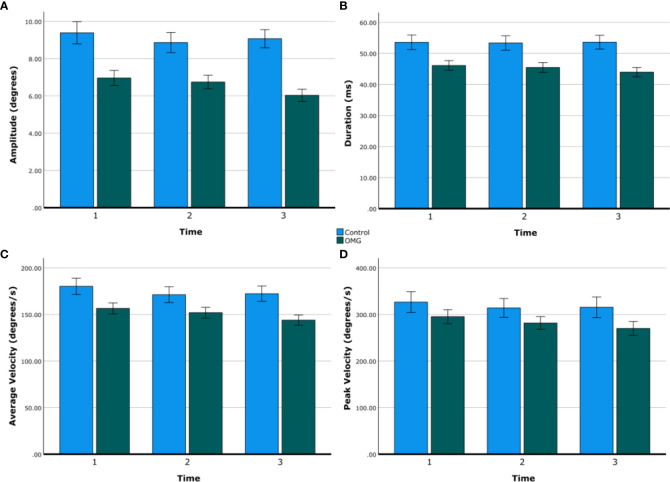
Saccadic metrics over time. Group means of saccadic amplitude **(A)**, duration **(B)**, average velocity **(C)**, and peak velocity **(D)** over time (first, second, and third minute) in control subjects (blue) and OMG patients (green). Error bars are ±1 SE.

**Figure 1** shows group summary statistics of all saccadic metrics over time. Saccadic amplitude, duration, and average velocity were lower in OMG patients than in control subjects [6.5° ± 0.3° vs. 9.1° ± 0.5°, 45.2 ± 1.5 vs. 53.5 ± 2.2 ms, 164.0° ± 3.5° vs. 188.9° ± 7.1° per second, respectively; all *F*(2,30) > 5.9, *p *< 0.021]. Saccadic peak velocity did not statistically differ between groups [OMG: 282.3° per second ± 14.1° per second; control: 318.7° per second ± 21.0° per second; *F*(1,30) = 2.0, *p *= 0.161] but tended to be smaller in OMG patients than in control subjects. Saccadic amplitude, average velocity, and peak velocity decreased over time [all *F*(2,60) > 4.2, *p *< 0.02] but this decrease did not differ between groups [time × group: *F*(2,48) = 1.3, *p *= 0.287]. *Post-hoc* tests revealed statistical differences between the first and the third minute after adjustment for multiple comparisons (amplitude: 8.2° ± 0.4° vs. 7.5° ± 0.3°, *p *= 0.034; average velocity: 168.3° per second ± 5.3° per second vs. 158.1° per second ± 5.0° per second, *p *= 0.002; peak velocity: 310.9° per second ± 13.4° per second vs. 292.7° per second ± 13.3° per second, *p *= 0.010). No significant effect of time or interaction between time and group was found on saccadic duration [all *F*(2,60) < 1.7, *p *> 0.2].

**Figure 2:**
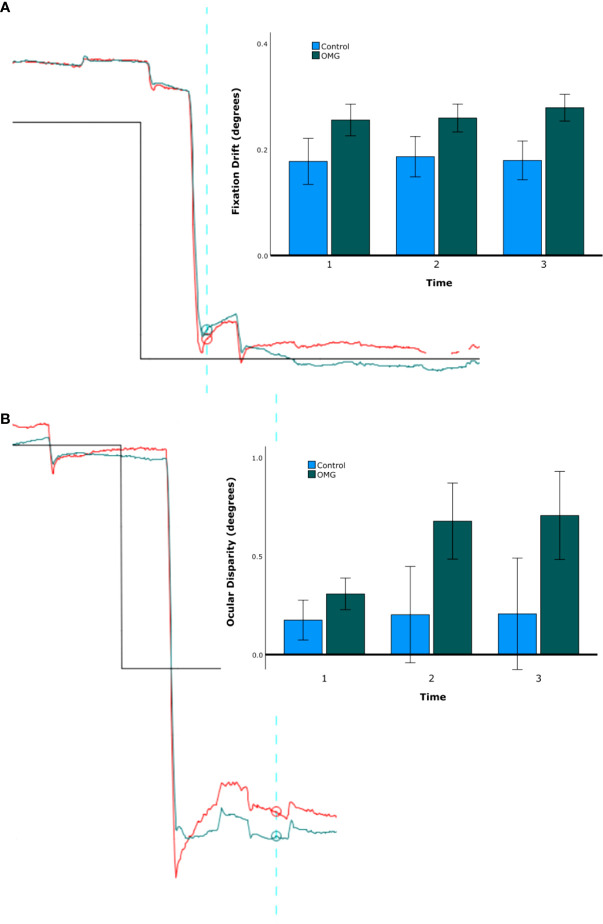
Fixation stability. Fixation drift **(A)** and ocular disparity **(B)**. The traces on the left-hand side of A and B shows the right (red) and left (green) gaze position (*y*-axis) over time (*x*-axis) for one representative OMG patient with abnormal signs and symptoms. The upward direction in the graph represents the leftward gaze, downward direction represents the rightward gaze. Note the difference in gaze position between the start (blue vertical line) and the end (furthest right time point) of fixation in A and the difference in gaze position between the right and the left eyes over time in **(B)**. Note also the hypometria with glissade, implying a pulse-step mismatch and a recognised feature of OMG (16). The bar charts on the right-hand side of A and B show group mean ( ± 1 SE) fixation stability over the three time bins (first, second, and third minute) for OMG patients (green) and control subjects (blue).

**Figure 2** shows example gaze traces and group summary statistics of fixation stability. Fixation drift and ocular disparity were generally greater in OMG patients than in control subjects (0.24° ± 0.02° vs. 0.16° ± 0.04°; 0.6° ± 0.1° vs. 0.2° ± 0.2°, respectively) but the differences were not statistically significant [*F*(1,29) = 3.3, *p *= 0.080, and *F*(1,24) = 2.7, *p *= 0.114, respectively]. No significant effects of time or group by time were found on fixation drift and ocular disparity (all *p *> 0.3).

**Figure 3:**
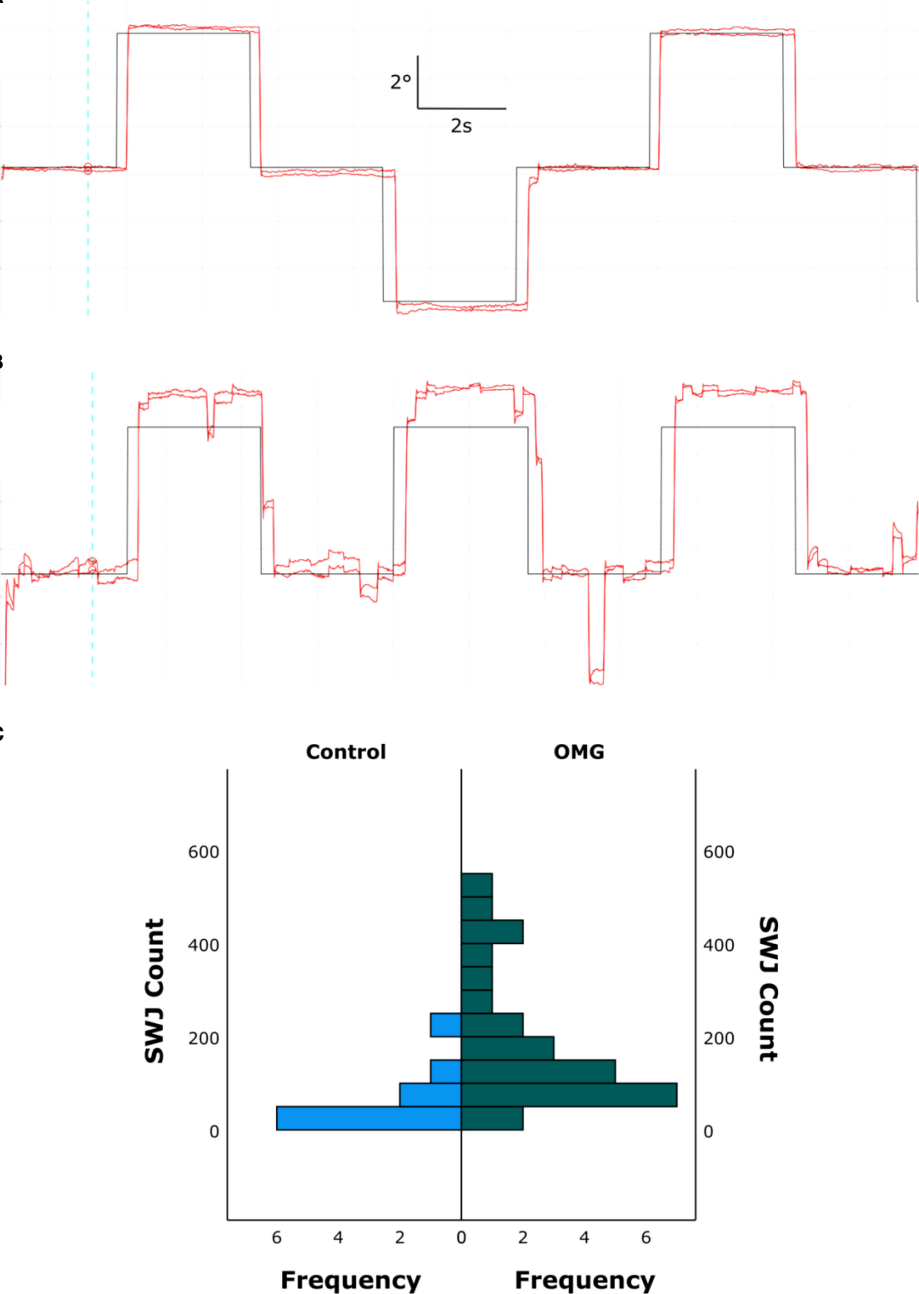
Square wave jerks. Binocular gaze position (red) over time in one control subject **(A)** and one OMG patient with abnormal signs and symptoms **(B)** making saccadic movements to targets at ±5° from the primary position. Target location over time is depicted in black. The upward direction in the graph represents leftward gaze, downward direction represents rightward gaze. Note the increased frequency of SWJs during fixation in B compared with A. **(C)** Histogram showing all counted SWJs for all control subjects (left, blue) and OMG patients (right, green).

The number of SWJs (median [IQR]); was significantly larger in OMG patients than in control subjects [OMG: 145.5 [84 272]; control: 32.5 [22 85] *U *= 223, *z *= 3.29, *p *< 0.001]. **Figure 3** shows an example of increased SWJ frequency for one control (**Figure 3A**) and OMG (**Figure 3B**) participant, and the distribution of all counted SWJs for each group (**Figure 3C**).

## Discussion

This preliminary study using state-of-the-art modern oculography adds to our knowledge of OMG, as only a few oculography studies have been conducted previously (6, 8, 10, 11, 17, 18). Overall, the number of saccadic intrusions during attempted steady fixation in patients with OMG was twice that of control subjects. OMG patients also showed a tendency to dysconjugate ocular drift between left and right eyes following large horizontal saccades; this has not, to our knowledge, been previously described in patients with this condition.

We also observed reduced saccadic amplitude and velocity in OMG patients compared with control subjects, in keeping with previous observations that slow-twitch (tonic) muscle fibres are preferentially affected in MG (8, 9, 11, 17). Saccades in the third minute were significantly smaller than those in the first minute of recording in OMG patients, consistent with fatigue, but velocity did not decrease. Unlike previous reports, we found saccadic velocities to be significantly lower in OMG patients than in control subjects (8), indicating at least some involvement of fast-twitch fibres. However, such fibres appear sufficient to generate “twitch” or “quiver” movements in the presence of severe ophthalmoplegia, a characteristic feature of MG (8). A recent study using video-nystagmography found a decrement in EOM activity in patients with MG that improved within 1–2 seconds after reaching minimum velocity (19). This is in keeping with our findings that MG affects saccades.

A range of saccadic intrusions were described by Smidt and colleagues in a study of 12 patients with OMG, including a saccadic pulse (stepless saccade) with an exponential decay back to the baseline, a double saccadic pulse, SWJs, macro-SWJs, and macro-saccadic oscillations (8). We did not systematically classify these in our study but noted a similar range of intrusions across OMG subtypes. Although saccadic intrusions are a feature of many cerebellar disorders, such as Friedreich’s ataxia (20), recent oculomotor models propose that they are the result of dysfunction within the brainstem ocular motor network (21), which includes excitatory burst neurons (EBNs), inhibitory burst neurons (IBNs), omnipause neurons (OPNs), and their connections with the superior colliculus (SC). Increased fluctuation of neural activity within the SC is thought to increase input to EBNs and decrease input to OPNs, leading to a short burst of activity in the EBNs that produces a small saccade (21). In turn, this produces a small retinal error that is detected in the SC network and results in a second saccade in the opposite direction, generating a SWJ. The cerebellar fastigial nucleus has direct projections to IBNs that may modify the brainstem ocular motor network. Saccadic intrusions in OMG may instead reflect centrally mediated attempts to correct fixational instability related to abnormal gaze holding (8). It has been suggested that micro-SWJs may be a physiological response to impaired gaze fixation, increasing in amplitude with larger fixation targets and in darkness (22). This could account for the presence of SWJs in healthy individuals (e.g. with fatigue or altered fixation target) and in neurological disorders with impaired tonic gaze holding (e.g. MG, brainstem disease, and cerebellar disease). Indeed, this is in accord with our observation that fixation drift tends to be increased in patients with OMG. Given a tendency for each eye to show a different degree of drift following a saccade, further research should explore differences in SWJ frequencies between the right and left eyes. Our objective finding of fixation drift and saccadic intrusions mirrors our anecdotal experience of patients with OMG who have symptomatic diplopia but in whom examination reveals only minimal ophthalmoplegia. Such intrusions may be a surrogate measure of central compensation for this gaze-holding deficit.

This preliminary study has a number of limitations. First, although we aimed to include patients with the range of OMG subtypes, there were insufficient numbers of patients with each subtype for us to carry out independent comparisons of subtypes. Second, we were able to recruit only 10 healthy control participants owing to the COVID-19 pandemic and constraints on healthy individuals attending clinical care settings during this time. Third, we were unable to systematically analyse the range of saccadic intrusions owing to the variability of intrusion waveforms between and within individuals with OMG. Instead, we opted to categorise saccadic intrusions based on other oculographic studies in patients with neurological disorders and the known characteristics of SWJs (23–25).

Future studies should further explore the characteristics of saccadic intrusions, with a focus on interocular differences and the timing of these intrusions relative to the saccadic movements (e.g. pre, per, or post). Moreover, our study was insufficiently powered to identify whether or not such oculomotor abnormalities differ between seropositive and seronegative OMG groups, and whether or not such findings correlate with anecdotal findings that seropositive OMG patients manifest more severe ophthalmoplegia than seronegative individuals who have more variable and less severe ophthalmoplegia.

## Conclusions

Here we report on one of the largest cohorts of OMG patients, exploring saccadic and fixational eye movement abnormalities. Our data on saccadic eye movements show that the fast-twitch fibres in OMG are affected, with amplitude, duration, and average velocity all reduced compared with normal control subjects. We found a trend towards increased saccadic intrusions and propose that these may reflect a central mechanism of compensation for gaze-holding deficits. Understanding the characteristics of these abnormalities and how they correlate with clinical features, such as diplopia, may help us further understand the pathophysiology of OMG and its impact on an individual’s day-to-day function.

## Data availability statement

The raw data supporting the conclusions of this article will be made available by the authors, without undue reservation.

## Ethics statement

The studies involving human participants were reviewed and approved by Wales REC 6 Research Ethics Committee. The patients/participants provided their written informed consent to participate in this study.

## Author contributions

SW – design of the study, data analysis, writing of the manuscript, and principal investigator for the study. MB – data analysis, and writing and review of the manuscript. VT-H – design of the study, data collection, and writing and review of the manuscript. MA – data collection and review of the manuscript. AK – data collection and review of the manuscript. CN – data collection and review of the manuscript. MT – design of the study and review of the manuscript. DK – design of the study, data analysis, and review of the manuscript. All authors contributed to the article and approved the submitted version.

## Funding

This study was funded by a grant from the Myaware Charity. DK is supported by the National Institute for Health Research University College London Hospitals Biomedical Research Centre.

## Acknowledgments

We are grateful to the Myaware charity for funding this study, and the National Institute of Health and Care Research for portfolio adoption within the Clinical Research Network. We thank Sam Hutton, SR Research, for extraordinary support. We thank Mr. M.Panju for participant feedback in the early development of this study, and all participants for their important contributions to this study. We also thank Kelly MacKenzie, Moorfields Eye Hospital head orthoptist, for her support of this study.

## Conflict of interest

The authors declare that the research was conducted in the absence of any commercial or financial relationships that could be construed as a potential conflict of interest.

## Publisher’s note

All claims expressed in this article are solely those of the authors and do not necessarily represent those of their affiliated organizations, or those of the publisher, the editors and the reviewers. Any product that may be evaluated in this article, or claim that may be made by its manufacturer, is not guaranteed or endorsed by the publisher.
